# Approach to Investigation of Hyperandrogenism in a Postmenopausal Woman

**DOI:** 10.1210/clinem/dgac673

**Published:** 2022-11-21

**Authors:** Angelica Lindén Hirschberg

**Affiliations:** Department of Women's and Children's Health, Karolinska Institutet, Stockholm SE-171 76, Sweden; Department of Gynecology and Reproductive Medicine, Karolinska University Hospital, Stockholm SE-171 76, Sweden

**Keywords:** hyperandrogenism, hirsutism, virilization, postmenopausal women, ovarian hyperthecosis, androgen-producing tumor

## Abstract

Postmenopausal hyperandrogenism is a condition caused by relative or absolute androgen excess originating from the ovaries and/or the adrenal glands. Hirsutism, in other words, increased terminal hair growth in androgen-dependent areas of the body, is considered the most effective measure of hyperandrogenism in women. Other symptoms can be acne and androgenic alopecia or the development of virilization, including clitoromegaly. Postmenopausal hyperandrogenism may also be associated with metabolic disorders such as abdominal obesity, insulin resistance, and type 2 diabetes. Mild hyperandrogenic symptoms can be due to relative androgen excess associated with menopausal transition or polycystic ovary syndrome, which is likely the most common cause of postmenopausal hyperandrogenism. Virilizing symptoms, on the other hand, can be caused by ovarian hyperthecosis or an androgen-producing ovarian or adrenal tumor that could be malignant. Determination of serum testosterone, preferably by tandem mass spectrometry, is the first step in the endocrine evaluation, providing important information on the degree of androgen excess. Testosterone >5 nmol/L is associated with virilization and requires prompt investigation to rule out an androgen-producing tumor in the first instance. To localize the source of androgen excess, imaging techniques are used, such as transvaginal ultrasound or magnetic resonance imaging (MRI) for the ovaries and computed tomography and MRI for the adrenals. Bilateral oophorectomy or surgical removal of an adrenal tumor is the main curative treatment and will ultimately lead to a histopathological diagnosis. Mild to moderate symptoms of androgen excess are treated with antiandrogen therapy or specific endocrine therapy depending on diagnosis. This review summarizes the most relevant causes of hyperandrogenism in postmenopausal women and suggests principles for clinical investigation and treatment.

## Case presentation

**Figure 1.**
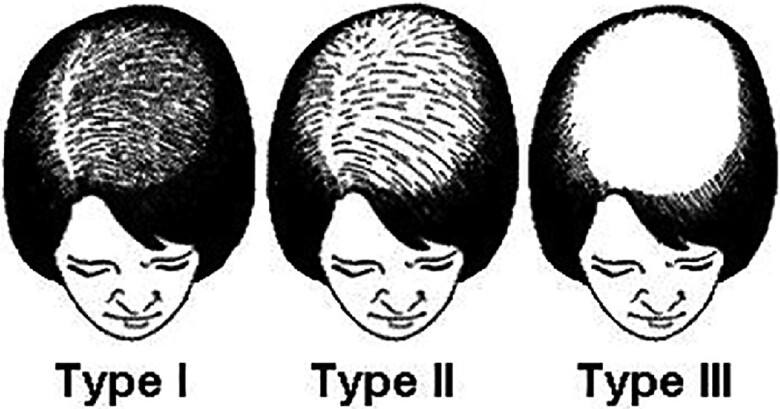
The Ludwig scale of androgen-dependent frontotemporal baldness type I to III, where type III is the most severe form of androgenic alopecia also called “Hippocratic baldness.”

A postmenopausal 66-year-old nulliparous woman with type 2 diabetes and hyperlipidemia is being referred to a specialist clinic at a university hospital due to suspected androgen-dependent hair loss that has developed over the years. She has frontotemporal baldness and has been using a wig for a couple of years. The woman first sought medical help many years ago but was told that it is normal with hair loss after menopause. When examining the patient, it is noted that she is overweight with body mass index (BMI) 29 and she has abdominal fat distribution. Furthermore, she has so-called “Hippocratic baldness,” corresponding to grade III on the Ludwig scale (1) (Fig. 1), oily skin, increased body hair, and blood pressure 160/90 mmHg. The most marked finding in laboratory analyses is a clearly elevated testosterone level of 5.6 nmol/L (Table 1). Furthermore, androstendione (A4) of 6.8 nmol/L is above normal for a postmenopausal woman (2). Dehydroepiandrosterone sulfate (DHEAS) (4.0 µmol/L) and sex hormone–binding globulin (SHBG) (28 nmol/L) are within the reference range. Gynecological examination shows clitoromegaly and a greatly enlarged uterus on palpation, as well as bilateral ovaries of significant size detected by transvaginal ultrasound. She is referred for a Doppler ultrasound examination, which confirms large uterine fibroids and enlarged ovaries with normal blood flow. The patient undergoes hysterectomy and bilateral salpingo-ophorectomy. Histopathological examination reveals benign uterine fibroids and bilateral ovarian stromal hyperplasia with the presence of nests of luteinized theca cells, in agreement with ovarian hyperthecosis. There are no signs of malignancy. Postoperatively, testosterone levels normalize within a couple of weeks (0.8 nmol/L). The symptoms subside spontaneously, resulting in weight loss, and reduced abdominal obesity, hirsutism, and oily skin. However, androgenic alopecia and clitoromegaly remain.

**Table 1.** Hormone values in the patient case

Hormone	Patient value	Reference interval
FSH, IU/L	30	30-150 postmenopausal
LH, IU/L	16	15-65 postmenopausal
Testosterone, nmol/L	5.6	0.3-3 premenopausal
SHBG, nmol/L	28	35-350
Androstendione, nmol/L	6.8	1.6-12 premenopausal
DHEAS, µmol/L	4.0	0.5-4.1
Estradiol, pmol/L	98	<40 postmenopausal

Abbreviations: DHEAS, dehydroepiandrosterone sulfate; FSH, follicle-stimulating hormone; LH, luteinizing hormone; SHBG, sex hormone–binding globulin.

## Menopausal Transition and Circulating Testosterone

Menopausal transition is associated with a decrease in the number of antral follicles and ovarian volume, as well as a decline in serum antimüllerian hormone (AMH) as a marker for antral follicle count and ovarian reserve. When the number of antral follicles and ovarian granulosa cells decrease, estradiol levels decline and follicle-stimulating hormone (FSH) levels increase. Menopause, the last spontaneous menstruation, occurs on average at age 51 years when circulating estradiol has decreased to a level insufficient to stimulate the endometrium to grow and then shed. During this period, menopausal symptoms, including hot flushes, sweating, and sleep problems, are common and associated with the gradual decline in estradiol.

In contrast to the decrease in estradiol, circulating levels of testosterone decline as a consequence of age-related, but not menopause-related, reductions in secretion by both the adrenal gland and the ovary (3). This means a 50% reduction in testosterone in women aged 40-45 compared with women in the 18-24 age group. In premenopausal women, about 50% of circulating testosterone arises from direct secretion from the ovary and the adrenal gland, in equal amounts, under pituitary control of luteinizing hormone (LH) and adrenocorticotropic hormone (ACTH), respectively (4). The remaining 50% of testosterone is produced from peripheral conversion by ovarian and adrenal inactive androgen precursors (A4, dehydroepiandrosterone (DHEA), and DHEAS). Testosterone is further converted in target tissues to dihydrotestosterone (DHT) by the enzyme 5α reductase, and together these hormones constitute the 2 classical bioactive androgens that bind to the androgen receptor. In postmenopausal women, a larger part of these active androgens is synthesized in peripheral tissue from DHEA within the cell according to the concept of intracrinology (5).

It was recently demonstrated that the androgen derivates 11-ketotestosterone and 11-ketodihydrotestosterone from the adrenal glands are also potent agonists of the human androgen receptor (6). In contrast to classical androgens (DHEA, DHEAS, A4, and testosterone), 11-keto androgens do not decrease with age (7). Furthermore, these androgens have shown to be predominant in several disorders of hyperandrogenism, including polycystic ovary syndrome (PCOS) (8) and congenital adrenal hyperplasia (CAH) (9). However, determination of 11-keto androgens is not yet available as a clinical routine method.

SHBG, a protein secreted by the liver, regulates the bioavailability of testosterone. Around 65% to 70% of circulating testosterone is bound and inactivated by SHBG, 30% to 35% is loosely bound to albumin, and only 0.5% to 3% represents freely circulating testosterone (10). Since the binding of testosterone to albumin is rather weak, the free and albumin-bound fractions are defined as bioavailable testosterone. The ratio of total testosterone to SHBG multiplied by 100 (the free androgen index) is used as a measure of circulating free testosterone. However, this measure is less relevant when total testosterone is pathologically increased.

During menopausal transition, ovarian theca cell production of testosterone decreases due to follicle depletion, but this loss is compensated by increased LH stimulation of stroma cell production of testosterone. Consequently, ovarian androgen production does not change significantly in relation to menopause. At the same time, SHBG decreases due to the decrease in ovarian estrogen production, and subsequently the free androgen index increases (11, 12). Overall, this will result in a physiological shift from estrogen dominance to a relative predominance of androgens during menopausal transition (13). Besides the typical menopausal symptoms, it is not uncommon for healthy postmenopausal women to experience androgen-dependent symptoms, such as increased facial hair growth and hair thinning due to relative androgen excess.

The most common cause of absolute androgen excess in postmenopausal women is PCOS, causing mild to moderate symptoms of hyperandrogenism (2), whereas virilizing symptoms including, for instance, clitoromegaly, deepening of the voice, and breast atrophy besides severe hirsutism and possibly androgenic alopecia (Table 2) are rare and should be carefully investigated. The most relevant causes of hyperandrogenism, of either ovarian or adrenal origin, in postmenopausal women are described below (Table 3).

**Table 2.** Mild to moderate and virilizing symptoms of hyperandrogenism in postmenopausal women

	Symptoms of hyperandrogenism
Mild to moderate symptoms	Hirsutism Acne and oily skin
Virilizing symptoms	Severe hirsutism and acne Androgenic alopecia Deepening of the voice Breast atrophy Increased muscle mass Enlargement of clitoris

**Table 3.** Characteristics of different conditions of hyperandrogenism in postmenopausal women

Condition	Prevalence/Incidence	Testosterone levels	Presentation
PCOS	Prevalence: 8% to 13% in the whole female population of fertile age	<2 nmol/L	Persistent or increased hirsutism but no virilizing symptoms History of oligo/amenorrhea and hyperandrogenism during reproductive years Later menopause Overweight, abdominal obesity
Ovarian hyperthecosis	Prevalence: 9.3% in postmenopausal women with hyperandrogenism (2)	>5 nmol/L	Gradual development of virilizing symptoms in a peri- or postmenopausal woman Isolated increase in testosterone Severe insulin resistance, acanthosis nigricans, metabolic syndrome, and/or type 2 diabetes Bilaterally enlarged ovaries
Androgen-secreting ovarian tumor	Prevalence: 2.7% in postmenopausal women with hyperandrogenism (2)	>5 nmol/L	Rapid onset of virilizing symptoms Serum testosterone often in the male range, accompanying increase in A4 and 17-OHP, but usually not DHEAS Unilateral ovarian tumor
Androgen-secreting adrenal tumor	Incidence: 1 to 2 cases/million population/year (14)	>5 nmol/L	Rapid onset of virilizing symptoms Serum testosterone in the male range, DHEAS, and cortisol usually elevated Unilateral adrenal tumor
Nonclassic congenital adrenal hyperplasia	Prevalence: 1% to 10% in women with hirsutism (15)	<5 nmol/L	Gradual increase in hirsutism since puberty Similar symptoms as in PCOS Serum testosterone moderately increased, 17-OHP elevated
Cushing's syndrome	Incidence: 1.8 to 3.2 cases/million population/year (16)	<2 nmol/L	New-onset hirsutism but seldom virilizing symptoms Typical signs of Cushing syndrome such as moon face, abdominal obesity, striae, buffalo hump ACTH-secreting pituitary adenoma or a cortisol-producing tumor

Abbreviations: 17-OHP, 17-hydroxyprogesterone; A4, androstenedione; ACTH, adrenocorticotropic hormone; DHEAS, dehydroepiandrosterone sulfate; PCOS, polycystic ovary syndrome.

## Etiology of Hyperandrogenism in Postmenopausal Women

### Polycystic Ovary Syndrome

PCOS is considered the most frequent endocrine disorder in women of reproductive age with a prevalence between 8% and 13% depending on diagnostic criteria and population studied (17, 18). According to the Rotterdam criteria, at least 2 of the following 3 criteria are required for a diagnosis: oligomenorrhea or amenorrhea; biochemical or clinical hyperandrogenism such as hirsutism and acne; and polycystic ovarian morphology (19). There are no specific criteria to diagnose PCOS after menopause. The Endocrine Society Clinical Practice Guideline has therefore suggested that a diagnosis of PCOS in a postmenopausal woman can be based upon a history of oligo/amenorrhea and hyperandrogenism during the reproductive years (20).

The reproductive phenotype of PCOS usually improves by age due to loss of ovarian follicles, leading to more regular cycles and decreased ovarian volume (21). However, the decrease in ovarian volume and serum AMH during menopausal transition may be relatively less in women with PCOS than in other women (22, 23). Consequently, the average age of menopause is approximately 2 years later in PCOS than in healthy controls (24). As androgen levels gradually decrease by age, symptoms of hyperandrogenism like hirsutism may improve in women with PCOS (25). Still, the prevalence of hirsutism was significantly higher in postmenopausal women with PCOS than in control women (33% vs 4%) at mean age 81 years in a Swedish long-term follow-up study (26).

PCOS is considered a relatively mild form of hyperandrogenism since circulating levels of testosterone usually are within the upper normal female range, whereas SHBG is low, resulting in increased levels of free and bioavailable testosterone. Today, liquid chromatography-tandem mass spectrometry (LC–MS/MS) is recognized as the gold standard method for testosterone determination in serum compared with immunobased clinical methods, which are burdened with cross-reactivity against structurally similar steroid hormones and, moreover, are not sensitive enough for the determination of steroids at relatively low concentrations (27). Available measurements based on LC–MS/MS indicate that the normal range of testosterone in premenopausal women is 0.1 to 1.8 nmol/L, whereas the upper limit in women with PCOS is 3.1 nmol/L (95% CI one-sided) (28). Although testosterone levels decline with increasing age, most studies have shown higher testosterone levels in postmenopausal women with PCOS than in control women (29-31). However, testosterone levels in postmenopausal women with PCOS seldom exceed 2 nmol/L (30-33).

PCOS is also a metabolic disorder with increased occurrence of obesity, which aggravates all symptoms of the syndrome, including hirsutism (34). Abdominal obesity is associated with insulin resistance, leading to secondary hyperinsulinemia (35). Hypersecretion of insulin stimulates ovarian androgen production in synergy with LH (35). In addition, insulin inhibits the hepatic synthesis of SHBG, leading to an increased free androgen index (36). In this way, obesity and insulin resistance contribute to hyperandrogenism in women with PCOS. Testosterone may in turn induce hepatic insulin resistance by facilitating catecholamine-stimulated lipolysis in visceral fat tissue, and peripheral insulin resistance in muscle tissue by inducing decreased capillary density (34, 37). Women with abdominal obesity often have a more pronounced PCOS phenotype and remain hyperandrogenic after menopause or may even have worsening symptoms. In the long run, PCOS is associated with an increased risk of type 2 diabetes and metabolic syndrome (18), whereas the risk of cardiovascular disease seems not to be increased after menopause (32).

Although hirsutism may be severe in PCOS, virilizing symptoms including clitoral enlargement are not associated with PCOS (Tables 2 and 3). In cases where hyperandrogenic symptoms increase and develop into virilization, other conditions of androgen excess must be ruled out.

### Ovarian Hyperthecosis

Ovarian hyperthecosis is a relatively rare disorder presenting with slow progress of severe symptoms of hyperandrogenism in a perimenopausal or postmenopausal woman (Table 3) (38). It is likely the second most frequent cause of hyperandrogenism in postmenopausal women. The prevalence of ovarian hyperthecosis was reported to be 9.3% in postmenopausal women undergoing investigation for symptoms of androgen excess (2).

**Figure 2.**
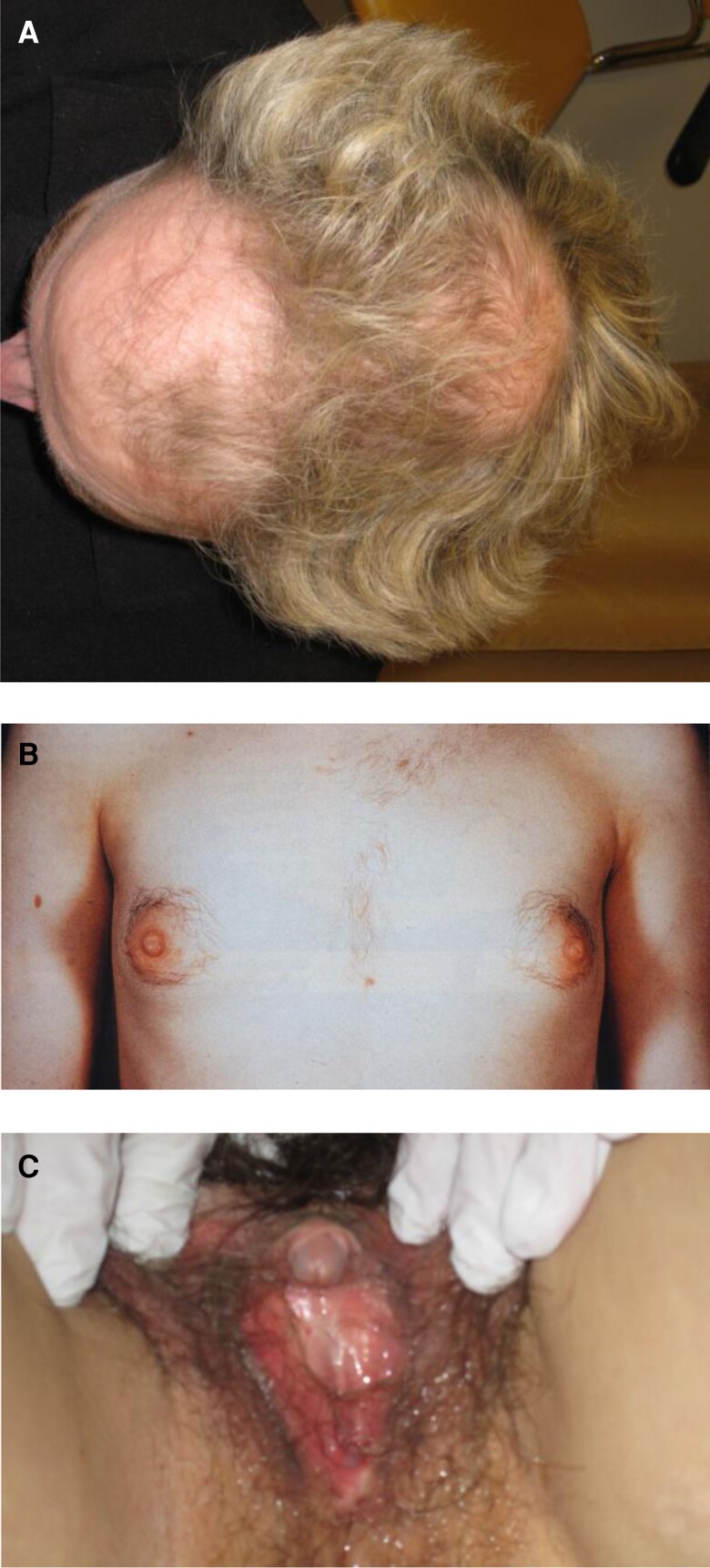
Clinical signs of severe hyperandrogenism and virilizing symptoms of hyperandrogenism in a perimenopausal and postmenopausal women including (A) androgenic alopecia, (B) breast atrophy and hirsutism, and (C) clitoromegaly.

The condition is often described as an extreme form of PCOS; however, there is no clear evidence of a link between ovarian hyperthecosis and PCOS, and most women with PCOS will never develop ovarian hyperthecosis. In contrast to PCOS, ovarian hyperthecosis will progress into virilizing symptoms, including severe hirsutism, androgenic alopecia, deepening of the voice, breast atrophy, and clitoromegaly (Table 2, Fig. 2). In addition, the ovaries are bilaterally clearly enlarged with a volume up to 10 cm^3^, compared with a volume between 1 and 5 cm^3^ of a normal postmenopausal ovary in women with or without PCOS (39-41). Ovarian hyperthecosis is strongly associated with metabolic symptoms, including abdominal obesity, hypertension, hyperlipidemia, insulin resistance, and acanthosis nigricans, in other words, the metabolic syndrome and type 2 diabetes (39, 42, 43). The metabolic symptoms are often more severe than in women with PCOS. Due to peripheral conversion of androgens to estrogens via aromatase, women with ovarian hyperthecosis also have an increased risk of endometrial pathology, including polyps, hyperplasia, and cancer (44, 45), as well as breast cancer (46).

Ovarian hyperthecosis is caused by nests of luteinized theca cells in the ovarian stroma producing high amounts of testosterone in the absence of other elevated androgens (47). Serum testosterone is usually increased above 5 nmol/L (38, 41), which distinguishes this condition from PCOS (Table 3). The etiology of ovarian hyperthecosis is not known, although a genetic disposition and association with PCOS have been suggested (48). Several mechanisms behind the increased testosterone production have been proposed. One is related to the “2-cell hypothesis” where ovarian testosterone production by theca cells is uncovered in a postmenopausal woman by the loss of granulosa cell–mediated aromatization of testosterone to estradiol (49). Another mechanism involves increased gonadotrophin stimulation by elevated levels of LH after menopause (50). Thirdly, there is support that insulin resistance and hyperinsulinemia may induce stromal luteinization causing androgen overproduction (51).

The most important differential diagnosis for ovarian hyperthecosis is an androgen-producing tumor, which can be malignant. In both cases, the patient has virilizing symptoms, but progress is usually slow for ovarian hyperthecosis but rapid for an androgen-producing tumor. In addition, testosterone is greatly elevated (>5 nmol/L) in both disorders, but mostly higher in women with an androgen-producing tumor than in those with ovarian hyperthecosis (41). Furthermore, other androgens are usually not elevated in ovarian hyperthecosis, whereas androgen-producing adrenal tumors are associated with high levels of DHEAS and A4, and ovarian tumors with high levels of inhibin B. Almost all women with ovarian hyperthecosis have obesity and insulin resistance, but this is not the case of women with an androgen-producing tumor. Ovarian hyperthecosis is also characterized by bilateral increase in ovarian stroma, whereas an ovarian tumor usually presents as a unilateral enlargement. Still, it can be difficult to distinguish ovarian hyperthecosis from an androgen-producing tumor. The diagnosis of ovarian hyperthecosis is therefore confirmed by histopathology.

### Androgen-Secreting Ovarian Tumors

Androgen-secreting ovarian tumors originate from sex cord stroma and include Sertoli cell tumors, Sertoli–Leydig cell tumors, Leydig cell tumors, thecoma, and granulosa cell tumors (52, 53). These tumors are predominantly benign and occur at any age, but approximately 25% present after menopause (54). Together they comprise 5% to 8% of all ovarian neoplasms (29). The presentation is often rapid progress of clinical manifestations of excessive androgen and/or estrogen production (Table 3). Androblastomas (Sertoli cell tumors, Sertoli–Leydig cell tumors, Leydig cell tumors) are those primarily secreting androgens, while thecoma and granulosa cell tumors mainly secrete estrogens, which can lead to postmenopausal bleeding, endometrial hyperplasia, or cancer. However, around 10% of granulosa cell tumors secrete androgens and may cause virilization (55). The prevalence of an androgen-secreting ovarian tumor in postmenopausal women with symptoms of hyperandrogenism has been reported to be 2.7% (2).

Endocrine characteristics of androblastomas are clearly elevated testosterone, often in the lower male range (8-29 nmol/L) (28), with an accompanying increase in A4 and 17-hydroxyprogesterone (17-OHP), whereas DHEAS and cortisol levels usually are normal (52). In the case of granulosa cell tumors, they usually cosecrete AMH and inhibin B, besides estradiol and/or testosterone (56, 57).

Ovarian tumors are often small but can be identified using transvaginal ultrasound with color Doppler or magnetic resonance imaging (MRI). Asymmetry of the ovaries may suggest a tumor.

### Androgen-Secreting Adrenal Tumors

Androgen-secreting adrenal tumors are less common than the corresponding ovarian tumors. Benign adrenal adenomas include nonsecretory (incidentalomas) and secretory adenomas, of which the latter can cause hyperandrogenism. Adrenocortical carcinomas, on the other hand, are usually highly malignant tumors, and approximately 25% of cases are associated with severe symptoms of hyperandrogenism leading to virilization (Table 3) (2, 58-60). The incidence is 1 to 2 cases/million population per year. There is a bimodal age distribution, with peaks before the age of 5 and in the fourth and fifth decade of life (14).

Adrenocortical carcinomas are considered gonadotropin independent and are manifested by an increase in adrenal androgens, DHEA, and DHEAS (2). DHEAS concentrations are often more than twice the upper limit, and a value above 19 µmol/L is an indication for further evaluation. Furthermore, testosterone is highly elevated in the male range, as is A4 (58, 59). Cortisol secretion may be increased both in androgen-secreting adenomas and carcinomas, leading to a clinical image of Cushing syndrome in addition to hyperandrogenism.

Adrenal tumors are best visualized by computed tomography (CT) as a unilateral mass. Adenomas are usually small, 2 to 2.5 cm, whereas adrenocortical carcinomas are larger, between 4 and 21 cm (60).

### Nonclassical Congenital Adrenal Hyperplasia

CAH is an autosomal recessive disease characterized by low or absent production of cortisol and aldosterone with concomitant overproduction of androgens due to an enzyme deficiency in the adrenal cortex steroid biosynthesis (61). The most common type is 21-hydroxylase deficiency caused by a mutation in the gene (*CYP21A1*) encoding the adrenal 21-hydroxylase enzyme (62). There are different clinical forms of CAH depending on the degree of enzyme deficiency: the severe salt wasting form (SW CAH), the simple virilizing form (SV CAH), and the less severe nonclassic form (NC CAH). SW CAH and SV CAH, often referred to as classic CAH, are usually diagnosed in infancy via newborn screening programs if available, due to varying degrees of virilization in females, or in the most severe cases due to life-threatening SW (62). The treatment of CAH consists of substitution therapy with glucocorticoids and mineralocorticoids, which will reduce the overproduction of androgens (62).

In contrast to classic CAH, women with NC CAH are usually diagnosed later in life due to mild symptoms of androgen excess, such as hirsutism, menstrual disorders, and infertility (Table 3) (15). The symptoms are very similar to PCOS, and, in agreement with PCOS, NC CAH is not associated with virilizing symptoms. Since some of these women may be undiagnosed or have worsening symptoms by age (63), NC CAH should be considered in postmenopausal women with hyperandrogenism. The NC form of CAH is estimated to be one of the most common autosomal recessive disorders, with a prevalence of 1% to 10% in women with hyperandrogenic symptoms (15).

Elevated serum 17-OHP, due to accumulation before the enzyme block, is indicative of CAH (64). It should be further investigated by an ACTH stimulation test. Serum concentrations of testosterone and adrenal androgen precursors (A4, DHEA, and DHEAS) are also increased. The diagnosis is confirmed by genetic testing and detection of a mutation causing enzyme deficiency and impaired corticosteroid synthesis.

### Cushing Syndrome

Cushing syndrome is a rare disorder, which can be either ACTH-dependent and caused by pituitary hypersecretion of ACTH (Cushing disease, about 70%) or ACTH independent due to adrenocortical adenoma or carcinoma (about 20%) (65). ACTH-dependent Cushing is associated with elevated ACTH levels causing bilateral adrenocortical hyperplasia and hypersecretion of cortisol. In contrast, ACTH-independent Cushing disease is related to suppressed ACTH secretion due to negative feedback by increased cortisol secretion (65). The overall incidence of Cushing syndrome is estimated to be 1.8 to 3.2 cases per million population (16). Cushing disease occurs mainly in women aged 25-45 years.

The major clinical manifestations of Cushing syndrome are moon face and facial plethora, abdominal obesity, striae, buffalo hump, proximal muscle weakness, bruising, hypertension, glucose intolerance, depression, and other neuropsychological symptoms (66). About 50% of women with Cushing syndrome also have symptoms of hyperandrogenism, such as hirsutism, due to adrenal androgen excess (A4, DHEA, and DHEAS) (Table 3) (67). Furthermore, the free androgen index is increased by endogenous hypercortisolism, probably due to a decrease in SHBG. However, signs of hyperandrogenism are usually mild to moderate and seldom lead to virilization.

The diagnosis of Cushing syndrome is established by hypersecretion of cortisol, as measured by 24-hour urinary free cortisol, late-night salivary cortisol, or the 1-mg dexamethasone suppression test (68). Further evaluation is needed to determine ACTH dependence or independence by measurement of plasma ACTH, as well as pituitary, adrenal, or ectopic etiology by using MRI of the pituitary and CT of the adrenal glands or other relevant imaging. First-line treatment is surgical removal of the ACTH- or cortisol-secreting tumor (68).

### Iatrogenic

Iatrogenic causes of hyperandrogenism due to overuse or abuse of androgenic drugs should be considered. Systemic testosterone and DHEA treatment of hypoactive sexual desire disorder or other androgen deficiency–related symptoms in postmenopausal women may lead to overtreatment if not carefully monitored by measurement of serum testosterone (69). Furthermore, treatment with the antiepileptic drug valproic acid has been shown to increase the risk of a PCOS-like phenotype in epidemiological studies (70). The mechanism is attributed to direct stimulation of ovarian androgen production by valproic acid (71). The anabolic steroid danazol, previously used for treatment of endometriosis and still used as therapy for hereditary angioedema, has been reported to induce hirsutism (72). It is well known that anabolic steroids can cause virilization in women when abused (73).

## Evaluation

### Clinical Symptoms

**Figure 3.**
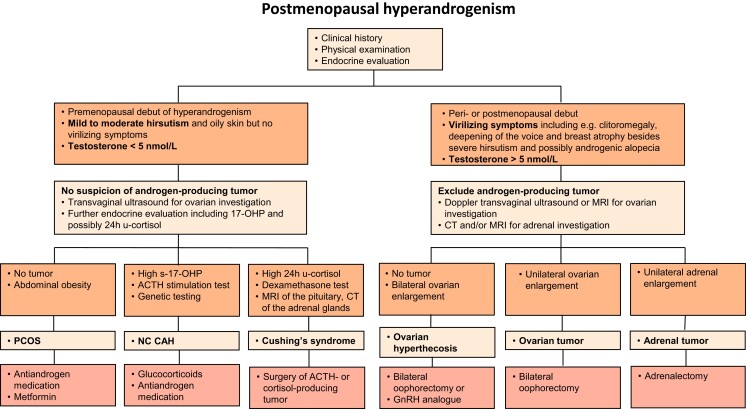
Algorithm for principles of investigation and treatment of different causes of hyperandrogenism in postmenopausal women. The cut-off of 5 nmol/L for serum testosterone is based on LC–MS/MS measurement. ACTH, adrenocorticotropic hormone; CT, computed tomography; GnRH, gonadotropin-releasing hormone; 17-OHP, 17-hydroxyprogesterone; MRI, magnetic resonance imaging; NC CAH, nonclassical congenital adrenal hyperplasia; PCOS, polycystic ovary syndrome.

The patient's history of onset and development of symptoms should always be a guide for further investigation. Late onset and rapid development of virilizing symptoms suggest a hormone-producing tumor, whereas slow development of virilizing symptoms in a perimenopausal or postmenopausal woman is typical of ovarian hyperthecosis. In contrast, early symptom onset and slow progression of mild to moderate hyperandrogenic symptoms are more consistent with PCOS or another endocrine disorder (Fig. 3).

Clinical signs of mild to virilizing symptoms of hyperandrogenism are shown in Table 2. Hirsutism has been considered the most effective measure of androgen excess in women (74). It is defined as excessive facial and body terminal hair in androgen-dependent body areas. Evaluation of hirsutism can be assessed by the modified Ferriman–Gallwey score, 0 (no terminal hair) to 4 (marked hirsutism), in 9 body areas: upper lip, chin and cheeks, upper chest, upper abdomen, lower abdomen, upper arms, thighs, upper back, and lower back (Table 4) (75, 76). A cut-off score of ≥ 4 to 6 on the modified Ferriman–Gallwey was suggested to indicate hirsutism, depending on ethnicity (77). However, this method has not been validated in postmenopausal women.

**Table 4.** Investigation of hyperandrogenism in postmenopausal women

	Assessment
Clinical symptoms	Hirsutism: modified Ferriman–Gallwey score Acne Androgenic alopecia: Ludwig score Clitoromegaly: >1.5 × 2.5 cm
Endocrine evaluation	Serum analyses of FSH, LH, testosterone, SHBG, A4, DHEAS, estradiol, 17-OHP, and inhibin B HOMA index or HbA1C as marker for insulin resistance can be considered ACTH stimulation test to rule out NC CAH 24-hour urinary free cortisol and dexamethasone test to rule out Cushing's syndrome
Imaging	Transvaginal ultrasound MRI, CT

Abbreviations: 17-OHP, 17-hydroxyprogesterone; A4, androstenedione; ACTH, adrenocorticotropic hormone; CT, computed tomography; DHEAS, dehydroepiandrosterone sulfate; FSH, follicle-stimulating hormone; HOMA, Homeostatic Model Assessment; LH, luteinizing hormone; MRI, magnetic resonance imaging; NC CAH, nonclassical congenital adrenal hyperplasia; SHBG, sex hormone–binding globulin.

Acne is associated with increased androgen levels, although the predictive value of acne for hyperandrogenism has been questioned (74, 78). Furthermore, there is no universally accepted classification tool for evaluation of acne (74). It is recommended to assess acne in a patient undergoing evaluation of hyperandrogenism (Table 3) (74).

Androgenic alopecia, or female pattern hair loss, is characterized by thinning of hair in the frontoparietal region of the scalp (Fig. 2) (79). The disorder is dependent on androgens, particularly DHT, and 5α reductase activity in hair follicles (79). Hair loss on the scalp can be assessed using the Ludwig scale (Table 4) (Fig. 1) (1).

Clitoromegaly is probably the most recognizable sign of virilization (Fig. 2). It has been defined as >1.5 × 2.5 cm (29, 80). However, signs of clitoromegaly must be carefully investigated, as they are easy to miss, especially in an obese woman. Other signs of virilization may be breast atrophy and severe hirsutism (Fig. 2).

### Endocrine Evaluation

Symptoms of hyperandrogenism and particularly virilizing symptoms (including, eg, clitoromegaly, deepening of the voice, and breast atrophy besides severe hirsutism and possibly androgenic alopecia) in a postmenopausal woman should be evaluated by endocrine screening, preferably serum FSH, LH, testosterone, SHBG, A4, DHEAS, estradiol, 17-OHP, and inhibin B (Table 4). For steroid hormone determination, it is highly recommended to use LC–MS/MS instead of immunological methods as mentioned above. Using receiver operating characteristic analysis, the diagnostic threshold for serum testosterone as measured by LC–MS/MS to identify an androgen-producing tumor was defined as testosterone ≥5.1 nmol/L (sensitivity, 90%; specificity, 81%) (33). Serum testosterone >5 nmol/L is clearly associated with virilizing symptoms (Table 2) (38, 81). It is therefore suggested to use this value of testosterone as the cut-off in the first step of investigation to rule out a hormone-producing tumor or a nontumor cause of severe hyperandrogenism such as ovarian hyperthecosis in a postmenopausal woman (Fig. 3).

#### Women with virilizing symptoms

Testosterone levels tend to be higher (in the lower male range) and gonadotropin levels lower in association with a virilizing ovarian tumor than in ovarian hyperthecosis; however, no cut-off value of testosterone has been proposed for discriminating between a hormone-producing tumor and ovarian hyperthecosis (29, 41, 82). The next step is therefore to proceed with diagnostic imaging (Fig. 3), see below. Further endocrine evaluation may still be helpful for distinguishing between tumorous and nontumorous causes of virilizing symptoms.

Ovarian hyperthecosis is typically associated with an isolated increase in testosterone, while other androgens usually are within the reference values (2), see the patient case above. Furthermore, insulin resistance is characteristic for ovarian hyperthecosis (39, 42, 43), and therefore fasting insulin and glucose for calculating the Homeostatic Model Assessment or HbA1c should be considered. In the case of hormone-producing ovarian tumors, hormones other than testosterone may also be elevated, including inhibin B, AMH, A4, 17-OHP, and estradiol, whereas DHEAS and cortisol are normal (52, 82). In contrast, androgen-producing adrenal tumors are associated with increased DHEAS levels, often higher than twice the upper normal limit (>19 µmol/L), together with increases in testosterone, A4, and cortisol (2).

The gonadotropin-releasing hormone (GnRH) agonist test can be used for distinguishing androgen-producing ovarian and adrenal tumors and successfully confirm an ovarian source from an adrenal source by suppression of testosterone (41). However, the test cannot differentiate between ovarian hyperthecosis and an ovarian tumor, as both disorders are gonadotropin dependent and will respond to GnRH with testosterone inhibition (83).

Selective ovarian and adrenal venous catheterization can localize an androgen-producing tumor by demonstrating differential gradients in androgen levels between ovarian, adrenal, and peripheral veins. However, success rates are poor, and this invasive method carries potential risks. A recent systematic review and meta-analysis concluded that there is limited evidence for the use of selective venous sampling in identifying androgen-producing tumors in postmenopausal women (84).

#### Women with mild to moderate symptoms of hyperandrogenism

In postmenopausal women with mild to moderate symptoms of hyperandrogenism and testosterone <5 nmol/L, PCOS is the most likely diagnosis, as supported by a premenopausal history of menstrual irregularities, hyperandrogenism, and/or polycystic ovaries (Fig. 3). In these women, androgen levels are slightly higher than in controls, but serum testosterone is in most cases below 2 nmol/L (30-33). Transvaginal ultrasound is recommended (see below) but further endocrine evaluation is not needed if screening samples are normal and the diagnosis is supported by medical history.

In the case of an increased morning value of 17-OHP (>9 nmol/L), the ACTH stimulation test should be performed to rule out NC CAH (Fig. 3) (62). A significant increase in ACTH in response to the test requires further investigation with genetic testing to confirm a CAH diagnosis (62). If Cushing syndrome is suspected because of its typical symptoms, this should be investigated with 24-hour urinary free cortisol and/or dexamethasone suppression test, as well as plasma ACTH followed by imaging, see below (Fig. 3).

### Imaging

Transvaginal ultrasound is used to investigate a possible ovarian cause of hyperandrogenism, primarily an androgen-producing ovarian tumor. The mean size of a normal postmenopausal ovary is estimated to be 2.2 ± 0.01 cm^3^ with a 95% upper CI of <5.0 cm^3^ (85). An ovarian tumor can be very small and difficult to identify, but asymmetry of the ovaries could be suggestive of such a tumor. Furthermore, color Doppler ultrasound can identify a hypervascularized region, suggesting a tumor (82). Failure to identify a tumor with ultrasound does not rule out this possibility; it is therefore important to investigate further with MRI (41, 82). Ovarian hyperthecosis is associated with bilateral homogeneous enlargement of the ovarian stroma on ultrasound without hypervascularization (40, 41). The average ovarian volume may be up to 10 cm^3^ (39-41). Postmenopausal women with PCOS usually have larger ovaries than control women, although significantly less than 10 cm^3^ (22).

MRI has shown a higher positive and negative predictive value (78%) and (100%), respectively, for detection of an androgen-secreting ovarian tumor than transvaginal ultrasound (82). In addition, good sensitivity (83%) and specificity (80%) were reported for MRI in differentiating between virilizing ovarian tumors and ovarian hyperthecosis (41).

CT is the preferable imaging technique for detecting adrenal tumors; it will detect nodules >5 mm (86). MRI could be an alternative imaging method for detecting adrenal tumors.

The majority of cases of Cushing syndrome are ACTH-dependent, and the next step of evaluation is to identify a possible pituitary tumor using MRI (68). If no mass is identified, petrosal sinus sampling and/or further imaging to identify an ectopic source of ACTH should be considered.

## Treatment

Treatment strategies depend on the cause, symptoms, and distress of androgen excess (Fig. 3). Patients with severe symptoms and testosterone >5 nmol/L should be managed urgently, especially those with suspected malignancy. A postmenopausal woman with virilizing symptoms and indication of an ovarian source of androgen excess (ovarian tumor, ovarian hyperthecosis) is primarily treated with surgery, in other words, laparoscopic bilateral oophorectomy, with or without hysterectomy (38, 41, 87). As in the patient case presented, testosterone levels will normalize rapidly within a couple of weeks after surgery while symptoms of androgen excess, including hirsutism and oily skin and acne, will gradually resolve. Although swelling of the clitoris may regress, clitoral hypertrophy usually persists. The same applies to androgenic alopecia; severe hair loss usually remains even after normalized testosterone levels. Deepening of the voice caused by androgen excess is also a symptom that does not regress after treatment. It is therefore important to diagnose at an early stage for prompt management of potentially severe causes of hyperandrogenism, although androgen-producing ovarian tumors are seldom malignant (52, 53), and to avoid persistent symptoms despite elimination of androgen excess. Metabolic symptoms may improve to some extent but usually do not disappear (43, 88).

When a patient is not a suitable candidate for surgery for a benign ovarian cause of severe hyperandrogenism, treatment with GnRH analogs is an alternative (39, 43, 89). Depending on the menopausal status of the patient, she may experience symptoms of estrogen withdrawal, and in this situation estrogen add-back therapy could be considered (90). Estrogen substitution is also important to consider during long-term treatment with GnRH analogs given the risk of accelerated bone loss (91).

The primary treatment for androgen-secreting adrenal tumors is adrenalectomy for histopathological diagnosis of malignant or benign tumor (92). Complete surgical resection can be sufficient therapy. In cases of malignancy, adjuvant therapy with mitotane, chemotherapy, or radiation may be used. Adrenal function must be closely monitored after surgery for detection of adrenal insufficiency or recurrent tumor (92).

Postmenopausal women with testosterone <5 nmol/L and a history of PCOS with persistent or aggravating hyperandrogenic symptoms are treated with antiandrogen therapy (93). There are different types of antiandrogens with various mechanisms of action and potential side-effects (94). Ovarian androgen overproduction can be inhibited by GnRH analogues as mentioned above; androgen effects can be blocked by androgen receptor blockers (spironolactone, cyproterone acetate, flutamide) or by a 5α reductase inhibitor (finasteride) blocking the conversion of testosterone to DHT, which is the most potent androgen in peripheral tissue. However, treatment of metabolic symptoms with metformin or other substances has limited effect on hyperandrogenic symptoms (95). The efficacy of antiandrogens for treatment of hirsutism is moderate (96), and it must be explained to the patient that it takes time before any treatment effect can be observed (at least 6 months) and that the effect disappears when treatment is stopped.

Postmenopausal women with NC CAH are usually treated with antiandrogens, similarly to PCOS, and cortisone is seldom needed (15). Cushing syndrome is primarily treated with surgery for an ACTH-producing pituitary or ectopic tumor or cortisol-secreting adrenal tumor (68). In some cases, medical therapy or radiation may be necessary (68).

## Conclusion

Postmenopausal hyperandrogenism ranges from mild symptoms due to relative androgen excess during menopausal transition to virilizing symptoms caused by a hormone-producing ovarian or adrenal tumor that could be malignant. A thorough investigation must be performed to determine the underlying cause and to offer appropriate treatment. Onset of symptoms, development, and severity of symptoms are indicative for further investigation. Measurement of serum testosterone, preferably by LC–MS/MS, provides important information on the degree of androgen excess. Testosterone >5 nmol/L is associated with virilizing symptoms and should prompt further investigation with imaging modalities to rule out an androgen-producing tumor. There is no discriminatory method to distinguish an ovarian hormone–producing tumor from ovarian hyperthecosis, although the latter is related to slower development of virilizing symptoms and bilateral ovarian enlargement. Surgery with bilateral oophorectomy or removal of an adrenal tumor is the main curative treatment and will ultimately lead to a histopathological diagnosis. GnRH analogs can be used as an alternative treatment of ovarian hyperthecosis. PCOS is probably the most common cause of mild to moderate symptoms of hyperandrogenism and testosterone <5 nmol/L in postmenopausal women. In this case, the recommended treatment is antiandrogen therapy using an androgen receptor blocker and/or a 5α reductase inhibitor. NC CAH could be treated in a similar way or if needed with cortisone, whereas Cushing syndrome is primarily treated with surgery.

## Financial support

No financial support for writing this paper.

## Disclosures

The author declares no conflicts of interest.

## Data Availability

Data sharing is not applicable to this article as no datasets were generated or analyzed during the current study.

## Abbreviations

17-OHP: 17-hydroxyprogesterone
A4: androstendione
ACTH: adrenocorticotropic hormone
AMH: antimüllerian hormone
BMI: body mass index
CAH: congenital adrenal hyperplasia
CT: computed tomography
DHEAS: dehydroepiandrosterone sulfate
DHT: dihydrotestosterone
FSH: follicle-stimulating hormone
GnRH: gonadotropin-releasing hormone
LC–MS/MS: liquid chromatography-tandem mass spectrometry
LH: luteinizing hormone
MRI: magnetic resonance imaging
NC: nonclassic
PCOS: polycystic ovary syndrome
SHGB: sex hormone–binding globulin
SV: simple virilizing
SW: salt wasting

## References

[1] Ludwig E . Classification of the types of androgenetic alopecia (common baldness) occurring in the female sex. Br J Dermatol. 1977;97(3):247‐254. 921894 10.1111/j.1365-2133.1977.tb15179.x

[2] Elhassan YS , Idkowiak J, Smith K, et al Causes, patterns, and severity of androgen excess in 1205 consecutively recruited women. J Clin Endocrinol Metab. 2018;103(3):1214‐1223. 29342266 10.1210/jc.2017-02426 PMC5868408

[3] Davison SL , Bell R, Donath S, Montalto JG, Davis SR. Androgen levels in adult females: changes with age, menopause, and oophorectomy. J Clin Endocrinol Metab. 2005;90(7):3847‐3853. 15827095 10.1210/jc.2005-0212

[4] Longcope C . Adrenal and gonadal androgen secretion in normal females. Clin Endocrinol Metab. 1986;15(2):213‐228. 10.1016/s0300-595x(86)80021-4 3013468

[5] Labrie F , Martel C, Belanger A, Pelletier G. Androgens in women are essentially made from DHEA in each peripheral tissue according to intracrinology. J Steroid Biochem Mol Biol. 2017;168(April):9‐18. 28153489 10.1016/j.jsbmb.2016.12.007

[6] Turcu AF , Rege J, Auchus RJ, Rainey WE. 11-Oxygenated androgens in health and disease. Nat Rev Endocrinol. 2020;16(5):284‐296. 32203405 10.1038/s41574-020-0336-x PMC7881526

[7] Nanba AT , Rege J, Ren J, Auchus RJ, Rainey WE, Turcu AF. 11-Oxygenated C19 steroids do not decline with age in women. J Clin Endocrinol Metab. 2019;104(7):2615‐2622. 30753518 10.1210/jc.2018-02527 PMC6525564

[8] O’Reilly MW , Kempegowda P, Jenkinson C, et al 11-Oxygenated C19 steroids are the predominant androgens in polycystic ovary syndrome. J Clin Endocrinol Metab. 2017;102(3):840‐848. 27901631 10.1210/jc.2016-3285 PMC5460696

[9] Turcu AF , Nanba AT, Chomic R, et al Adrenal-derived 11-oxygenated 19-carbon steroids are the dominant androgens in classic 21-hydroxylase deficiency. Eur J Endocrinol. 2016;174(5):601‐609. 26865584 10.1530/EJE-15-1181 PMC4874183

[10] Dunn JF , Nisula BC, Rodbard D. Transport of steroid hormones: binding of 21 endogenous steroids to both testosterone-binding globulin and corticosteroid-binding globulin in human plasma. J Clin Endocrinol Metab. 1981;53(1):58‐68. 7195404 10.1210/jcem-53-1-58

[11] Gershagen S , Doeber A, Jeppsson S, Rannevik G. Decreasing serum levels of sex hormone-binding globulin around the menopause and temporary relation to changing levels of ovarian steroids, as demonstrated in a longitudinal study. Fertil Steril. 1989;51(4):616‐621. 2924930 10.1016/s0015-0282(16)60609-x

[12] Burger HG , Dudley EC, Cui J, Dennerstein L, Hopper JL. A prospective longitudinal study of serum testosterone, dehydroepiandrosterone sulfate, and sex hormone-binding globulin levels through the menopause transition. J Clin Endocrinol Metab. 2000;85(8):2832‐2838. 10946891 10.1210/jcem.85.8.6740

[13] Torréns JI , Sutton-Tyrrell K, Zhao X, et al Relative androgen excess during the menopausal transition predicts incident metabolic syndrome in midlife women: study of women's health across the nation. Menopause. 2009;16(2):257‐264. 18971793 10.1097/gme.0b013e318185e249 PMC2950016

[14] Ng L , John M, Libertino JM. Adrenocortical carcinoma: diagnosis, evaluation and treatment. J Urol. 2003;169(1):5‐11. 12478091 10.1016/S0022-5347(05)64023-2

[15] Carmina E , Dewailly D, Escobar-Morreale HF, et al Non-classic congenital adrenal hyperplasia due to 21-hydroxylase deficiency revisited: an update with a special focus on adolescent and adult women. Hum Reprod Update. 2017;23(5):580‐599. 28582566 10.1093/humupd/dmx014

[16] Hakami OA , Ahmed S, Karavitaki N. Epidemiology and mortality of Cushing's syndrome. Best Pract Res Clin Endocrinol Metab. 2021;35(1):101521. 33766428 10.1016/j.beem.2021.101521

[17] Teede H , Deeks A, Moran L. Polycystic ovary syndrome: a complex condition with psychological, reproductive and metabolic manifestations that impacts on health across the lifespan. BMC Med. 2010;8(June 30):41. 20591140 10.1186/1741-7015-8-41 PMC2909929

[18] Azziz R , Carmina E, Chen Z, et al Polycystic ovary syndrome. Nat Rev Dis Primers. 2016;11(August 11):16057. 10.1038/nrdp.2016.57 27510637

[19] Rotterdam ESHRE/ASRM-Sponsored PCOS consensus workshop group . Revised 2003 consensus on diagnostic criteria and long-term health risks related to polycystic ovary syndrome (PCOS). Hum Reprod. 2004;19(1):41‐47. 14688154 10.1093/humrep/deh098

[20] Legro RS , Arslanian SA, Ehrmann DA, et al Diagnosis and treatment of polycystic ovary syndrome: an Endocrine Society clinical practice guideline. J Clin Endocrinol Metab. 2013;98(12):4565‐4592. 24151290 10.1210/jc.2013-2350 PMC5399492

[21] Elting MW , Kwee J, Korsen TJ, Rekers-Mombarg LT, Schoemaker J. Aging women with polycystic ovary syndrome who achieve regular menstrual cycles have a smaller follicle cohort than those who continue to have irregular cycles. Fertil Steril. 2003;79(5):1154‐1160. 12738511 10.1016/s0015-0282(03)00152-3

[22] Alsamarai S , Adams JM, Murphy MK, et al Criteria for polycystic ovarian morphology in polycystic ovary syndrome as a function of age. J Clin Endocrinol Metab. 2009;94(12):4961‐4970. 19846740 10.1210/jc.2009-0839 PMC2795657

[23] Brown ZA , Louwers YV, Fong SL, et al The phenotype of polycystic ovary syndrome ameliorates with aging. Fertil Steril. 2011;96(5):1259‐1265. 21963227 10.1016/j.fertnstert.2011.09.002

[24] Tehrani FR , Solaymani-Dodaran M, Hedayati M, Azizi F. Is polycystic ovary syndrome an exception for reproductive aging? Hum Reprod. 2010;25(7):1775‐1778. 20435693 10.1093/humrep/deq088

[25] Cooney LG , Dokras A. Beyond fertility: polycystic ovary syndrome and long-term health. Fertil Steril. 2018;110(5):794‐809. 30316414 10.1016/j.fertnstert.2018.08.021

[26] Forslund M , Schmidt J, Brännström M, Landin-Wilhelmsen K, Dahlgren E. Reproductive hormones and anthropometry: a follow-up of PCOS and controls from perimenopause to older than 80 years. J Clin Endocrinol Metab. 2021;106(2):421‐430. 33205205 10.1210/clinem/dgaa840

[27] Handelsman DJ . Mass spectrometry, immunoassay and valid steroid measurements in reproductive medicine and science. Hum Reprod. 2017;32(6):1147‐1150. 28453777 10.1093/humrep/dex078

[28] Handelsman DJ , Hirschberg AL, Bermon S. Circulating testosterone as the hormonal basis of sex differences in athletic performance. Endocr Rev. 2018;39(5):803‐829. 30010735 10.1210/er.2018-00020 PMC6391653

[29] Markopoulos MC , Kassi E, Alexandraki KI, Mastorakos G, Kaltsas G. Hyperandrogenism after menopause. Eur J Endocrinol. 2015;172(2):R79‐R91. 25225480 10.1530/EJE-14-0468

[30] Schmidt J , Brannstrom M, Landin-Wilhelmsen K, Dahlgren E. Reproductive hormone levels and anthropometry in postmenopausal women with polycystic ovary syndrome (PCOS): a 21-year follow-up study of women diagnosed with PCOS around 50 years ago and their age-matched controls. J Clin Endocrinol Metab. 2011;96(7):2178‐2185. 21508129 10.1210/jc.2010-2959

[31] Markopoulos M , Rizos D, Valsamakis G, et al Hyperandrogenism in women with polycystic ovary syndrome persists after menopause. J Clin Endocrinol Metab. 2011;96(3):623‐631. 21177795 10.1210/jc.2010-0130

[32] Meun C , Franco OH, Dhana K, et al High androgens in postmenopausal women and the risk for atherosclerosis and cardiovascular disease: The Rotterdam Study. J Clin Endocrinol Metab. 2018;103(4):1622‐1630. 29408955 10.1210/jc.2017-02421

[33] Sharma A , Kapoor E, Singh RJ, Chang AY, Erickson D. Diagnostic thresholds for androgen-producing tumors or pathologic hyperandrogenism in women by use of total testosterone concentrations measured by liquid chromatography-tandem mass spectrometry. Clin Chem. 2018;64(11):1636‐1645. 30068692 10.1373/clinchem.2018.290825

[34] Hirschberg AL . Polycystic ovary syndrome, obesity and reproductive implications. Womens Health (Lond). 2009;5(5):529‐540; quiz 541-542. 19702452 10.2217/whe.09.39

[35] Diamanti-Kandarakis E , Dunaif A. Insulin resistance and the polycystic ovary syndrome revisited: an update on mechanisms and implications. Endocr Rev. 2012;33(6):981‐1030. 23065822 10.1210/er.2011-1034 PMC5393155

[36] Xing C , Zhang J, Zhao H, He B. Effect of sex hormone-binding globulin on polycystic ovary syndrome: mechanisms, manifestations, genetics, and treatment. Int J Womens Health. 2022;14(February 2):91‐105. 35140526 10.2147/IJWH.S344542 PMC8818772

[37] Ek I , Arner P, Rydén M, et al A unique defect in the regulation of visceral fat cell lipolysis in the polycystic ovary syndrome as an early link to insulin resistance. Diabetes. 2002;51(2):484‐492. 11812759 10.2337/diabetes.51.2.484

[38] Meczekalski B , Szeliga A, Maciejewska-Jeske M, et al Hyperthecosis: an underestimated nontumorous cause of hyperandrogenism. Gynecol Endocrinol. 2021;37(8):677‐682. 33759685 10.1080/09513590.2021.1903419

[39] Krug E , Berga SL. Postmenopausal hyperthecosis: functional dysregulation of androgenesis in climacteric ovary. Obstet Gynecol. 2002;99(5 Pt 2):893‐897. 11975949 10.1016/s0029-7844(01)01588-5

[40] Rousset P , Gompel A, Christin-Maitre S, et al Ovarian hyperthecosis on grayscale and color Doppler ultrasound. Ultrasound Obstet Gynecol. 2008;32(5):694‐699. 18792416 10.1002/uog.6131

[41] Yance VRV , Marcondes JAM, Rocha MP, et al Discriminating between virilizing ovary tumors and ovary hyperthecosis in postmenopausal women: clinical data, hormonal profiles and image studies. Eur J Endocrinol. 2017;177(1):93‐102. 28432270 10.1530/EJE-17-0111

[42] Nagamani M , Van Dinh T, Kelver ME. Hyperinsulinemia in hyperthecosis of the ovaries. Am J Obstet Gynecol. 1986;154(2):384‐389. 3511712 10.1016/0002-9378(86)90676-9

[43] Barth JH , Jenkins M, Belchetz PE. Ovarian hyperthecosis, diabetes and hirsuties in post-menopausal women. Clin Endocrinol. 1997;46(2):123‐128. 10.1046/j.1365-2265.1997.1050916.x 9135690

[44] Nagamani M , Hannigan EV, Dinh TV, Stuart CA. Hyperinsulinemia and stromal luteinization of the ovaries in postmenopausal women with endometrial cancer. J Clin Endocrinol Metab. 1988;67(1):144‐148. 3288650 10.1210/jcem-67-1-144

[45] Zhang C , Sung CJ, Quddus MR, Simon RA, Jazaerly T, Lawrence WD. Association of ovarian hyperthecosis with endometrial polyp, endometrial hyperplasia, and endometrioid adenocarcinoma in postmenopausal women: a clinicopathological study of 238 cases. Hum Pathol. 2017;59(January):120‐124. 27746268 10.1016/j.humpath.2016.09.021

[46] Secreto G , Girombelli A, Krogh V. Androgen excess in breast cancer development: implications for prevention and treatment. Endocr Relat Cancer. 2019;26(2):R81‐R94. 30403656 10.1530/ERC-18-0429

[47] Brown DL , Henrichsen TL, Clayton AC, Hudson SB, Coddington CC III, Vella A. Ovarian stromal hyperthecosis: sonographic features and histologic associations. J Ultrasound Med. 2009;28(5):587‐593. 19389897 10.7863/jum.2009.28.5.587

[48] Fienberg R . Familial ovarian hyperthecosis. Am J Obstet Gynecol. 1972;112(2):309‐310. 10.1016/0002-9378(72)90137-8 4332765

[49] Nagamani M , Urban RJ. Increased expression of messenger ribonucleic acid encoding cytochrome P450 cholesterol side-chain cleavage and P450 17α-hydroxylase enzymes in ovarian hyperthecosis. Fertil Steril. 1999;71(2):328‐333. 9988407 10.1016/s0015-0282(98)00464-6

[50] Adashi EY . The climacteric ovary as a functional gonadotropin-driven androgen-producing gland. Fertil Steril. 1994;62(1):20‐27. 8005293 10.1016/s0015-0282(16)56810-1

[51] Brown RJ , Joseph J, Cochran E, et al Type B insulin resistance masquerading as ovarian hyperthecosis. J Clin Endocrinol Metab. 2017;102(6):1789‐1791. 27911591 10.1210/jc.2016-3674 PMC5470776

[52] Fleckenstein G , Sattler B, Hinney B, Wuttke W, Osmers R, Emons G. Androblastoma of the ovary: clinical, diagnostic and histopathologic features. Onkologie. 2001;24(3):286‐291. 11455224 10.1159/000055094

[53] Macut D , Ilić D, Jovanović AM, Bjekić-Macut J. Androgen-secreting ovarian tumors. Front Horm Res. 2019;53(September 9):100‐107. 31499493 10.1159/000494906

[54] Young RH , Scully RE. Ovarian Sertoli-Leydig cell tumors. A clinicopathological analysis of 207 cases. Am J Surg Pathol. 1985;9(8):543‐569. 3911780 10.1097/00000478-198508000-00001

[55] Sekkate S , Kairouani M, Serji B, et al Ovarian granulosa cell tumors: a retrospective study of 27 cases and a review of the literature. World J Surg Oncol. 2013;11(June 18):142. 23777285 10.1186/1477-7819-11-142 PMC3691822

[56] Healy DL , Burger HG, Mamers P, et al Elevated serum inhibin concentrations in postmenopausal women with ovarian tumors. New Engl J Med. 1993;329(21):1539‐1542. 8413476 10.1056/NEJM199311183292104

[57] Rey R , Sabourin JC, Venara M, et al Anti-Müllerian hormone is a specific marker of Sertoli- and granulosa-cell origin in gonadal tumors. Hum Pathol. 2000;31(10):1202‐1208. 11070112 10.1053/hupa.2000.18498

[58] Cordera F , Grant C, van Heerden J, Thompson G, Young W. Androgen-secreting adrenal tumors. Surgery. 2003;134(6):874‐880. 14668717 10.1016/s0039-6060(03)00410-0

[59] Moreno S , Montoya G, Armstrong J, et al Profile and outcome of pure androgen-secreting adrenal tumors in women: experience of 21 cases. Surgery. 2004;136(6):1192‐1198. 15657575 10.1016/j.surg.2004.06.046

[60] Angelousi A , Kassi E, Kaltsas GA. Current issues in the diagnosis and management of adrenocortical carcinomas. August 30 2021. In: Feingold KR, Anawalt B, Boyce A, et al, editors. Endotext [Internet]. MDText.com, Inc.; 2000. 25905240

[61] El-Maouche D , Arlt W, Merke DP. Congenital adrenal hyperplasia. Lancet. 2017;390(10108):2194‐2210. 28576284 10.1016/S0140-6736(17)31431-9

[62] Speiser PW , Arlt W, Auchus RJ, et al Congenital adrenal hyperplasia due to steroid 21-hydroxylase deficiency: an endocrine society* clinical practice guideline. J Clin Endocrinol Metab. 2018;103(11):4043‐4088. 30272171 10.1210/jc.2018-01865 PMC6456929

[63] Moran C , Azziz R, Carmina E, et al 21-Hydroxylase-deficient nonclassic adrenal hyperplasia is a progressive disorder: a multicenter study. Am J Obstet Gynecol. 2000;183(6):1468‐1474. 11120512 10.1067/mob.2000.108020

[64] Azziz R , Sanchez LA, Knochenhauer ES, et al Androgen excess in women: experience with over 1000 consecutive patients. J Clin Endocrinol Metab. 2004;89(2):453‐462. 14764747 10.1210/jc.2003-031122

[65] Lacroix A , Feelders RA, Stratakis CA, Nieman LK. Cushing's syndrome. Lancet. 2015;386(9996):913‐927. 26004339 10.1016/S0140-6736(14)61375-1

[66] Nieman LK . Cushing's syndrome: update on signs, symptoms and biochemical screening. Eur J Endocrinol. 2015;173(4):M33‐M38. 26156970 10.1530/EJE-15-0464 PMC4553096

[67] Kaltsas GA , Korbonits M, Isidori AM, et al How common are polycystic ovaries and the polycystic ovarian syndrome in women with Cushing's Syndrome? Clin Endocrinol. 2000;53(4):493‐500. 10.1046/j.1365-2265.2000.01117.x 11012575

[68] Nieman LK , Biller BM, Findling JW, et al The diagnosis of Cushing's syndrome: an Endocrine Society Clinical Practice Guideline. J Clin Endocrinol Metab. 2008;93(5):1526‐1540. 18334580 10.1210/jc.2008-0125 PMC2386281

[69] Martin KA , Anderson RR, Chang RJ, et al Evaluation and treatment of hirsutism in premenopausal women: an Endocrine Society Clinical Practice Guideline. J Clin Endocrinol Metab. 2018;103(4):1233‐1257. 29522147 10.1210/jc.2018-00241

[70] Hu X , Wang J, Dong W, Fang Q, Hu L, Liu C. A meta-analysis of polycystic ovary syndrome in women taking valproate for epilepsy. Epilepsy Res. 2011;97(1-2):73‐82. 21820873 10.1016/j.eplepsyres.2011.07.006

[71] Nelson-DeGrave VL , Wickenheisser JK, Cockrell JE, et al Valproate potentiates androgen biosynthesis in human ovarian theca cells. Endocrinology. 2004;145(2):799‐808. 14576182 10.1210/en.2003-0940

[72] Zotter Z , Veszeli N, Csuka D, Varga L, Farkas H. Frequency of the virilising effects of attenuated androgens reported by women with hereditary angioedema. Orphanet J Rare Dis. 2014;9(December 5):205. 25475444 10.1186/s13023-014-0205-6 PMC4268897

[73] Vorona E , Nieschlag E. Adverse effects of doping with anabolic androgenic steroids in competitive athletics, recreational sports and bodybuilding. Minerva Endocrinol. 2018;43(4):476‐488. 29463075 10.23736/S0391-1977.18.02810-9

[74] Lizneva D , Gavrilova-Jordan L, Walker W, Azziz R. Androgen excess: investigations and management. Best Pract Res Clin Obstet Gynaecol. 2016;37(November):98‐118. 27387253 10.1016/j.bpobgyn.2016.05.003

[75] Ferriman D , Gallwey JD. Clinical assessment of body hair growth in women. J Clin Endocrinol Metab. 1961;21(November):1440‐1447. 13892577 10.1210/jcem-21-11-1440

[76] Yildiz BO , Bolour S, Woods K, Moore A, Azziz R. Visually scoring hirsutism. Hum Reprod Update. 2010;16(1):51‐64. 19567450 10.1093/humupd/dmp024 PMC2792145

[77] Teede HJ , Misso ML, Costello MF, et al ; international PCOS network. Recommendations from the international evidence-based guideline for the assessment and management of polycystic ovary syndrome. Fertil Steril. 2018;110(3):364‐379. 30033227 10.1016/j.fertnstert.2018.05.004 PMC6939856

[78] Uysal G , Sahin Y, Unluhizarci K, et al Is acne a sign of androgen excess disorder or not? Eur J Obstet Gynecol Reprod Biol. 2017;211(April):21‐25. 28178574 10.1016/j.ejogrb.2017.01.054

[79] Price VH . Androgenetic alopecia in women. J Investig Dermatol Symp Proc. 2003;8(1):24‐27. 10.1046/j.1523-1747.2003.12168.x 12894991

[80] Tagatz GE , Kopher RA, Nagel TC, Okagaki T. The clitoral index: a bioassay of androgenic stimulation. Obstet Gynecol. 1979;54(5):562‐564. 503381

[81] Zaman A , Rothman MS. Postmenopausal hyperandrogenism: evaluation and treatment strategies. Endocrinol Metab Clin North Am. 2021;50(1):97‐111. 33518189 10.1016/j.ecl.2020.12.002 PMC9004339

[82] Sarfati J , Bachelot A, Coussieu C, et al Impact of clinical, hormonal, radiological, and immunohistochemical studies on the diagnosis of postmenopausal hyperandrogenism. Eur J Endocrinol. 2011;165(5):779‐788. 21896622 10.1530/EJE-11-0542

[83] Goyal A , Kubihal S, Gupta Y, Jyotsna VP, Khadgawat R. Dynamic testing for evaluation of adrenal and gonadal function in pediatric and adult endocrinology: an overview. Indian J Endocrinol Metab. 2019c;23(6):593‐601. 32042694 10.4103/ijem.IJEM_553_19 PMC6987775

[84] Tng EL , Tan JM. Dexamethasone suppression test versus selective ovarian and adrenal vein catheterization in identifying virilizing tumors in postmenopausal hyperandrogenism - a systematic review and meta-analysis. Gynecol Endocrinol. 2021;37(7):600‐608. 33660585 10.1080/09513590.2021.1897099

[85] Pavlik EJ , DePriest PD, Gallion HH, et al Ovarian volume related to age. Gynecol Oncol. 2000;77(3):410‐412. 10831351 10.1006/gyno.2000.5783

[86] Cavlan D , Bharwani N, Grossman A. Androgen- and estrogen-secreting adrenal cancer. Semin Oncol. 2010;37(6):638‐648. 21167382 10.1053/j.seminoncol.2010.10.016

[87] Mamoojee Y , Ganguri M, Taylor N, Quinton R. Clinical case seminar: postmenopausal androgen excess-challenges in diagnostic work-up and management of ovarian thecosis. Clin Endocrinol (Oxf). 2018;88(1):13‐20. 28980338 10.1111/cen.13492

[88] Pelusi C , Forlani G, Zanotti L, Gambineri A, Pasquali R. No metabolic impact of surgical normalization of hyperandrogenism in postmenopausal women with ovarian androgen-secreting tumours. Clin Endocrinol (Oxf). 2013;78(4):533‐538. 22583337 10.1111/j.1365-2265.2012.04438.x

[89] Vollaard ES , van Beek AP, Verburg FAJ, Roos A, Land JA. Gonadotropin-releasing hormone agonist treatment in postmenopausal women with hyperandrogenism of ovarian origin. J Clin Endocrinol Metab. 2011;96(5):1197‐1201. 21307133 10.1210/jc.2010-1991

[90] Della Corte L , Barra F, Mercorio A, et al Tolerability considerations for gonadotropin-releasing hormone analogues for endometriosis. Expert Opin Drug Metab Toxicol. 2020;16(9):759‐768. 32597340 10.1080/17425255.2020.1789591

[91] Sauerbrun-Cutler MT , Alvero R. Short- and long-term impact of gonadotropin-releasing hormone analogue treatment on bone loss and fracture. Fertil Steril. 2019;112(5):799‐803. 31731934 10.1016/j.fertnstert.2019.09.037

[92] Fassnacht M , Dekkers O, Else T, et al European Society of Endocrinology Clinical Practice Guidelines on the management of adrenocortical carcinoma in adults, in collaboration with the European Network for the Study of *Adrenal Tumors*. Eur J Endocrinol. 2018;179(4):G1‐G46. 30299884 10.1530/EJE-18-0608

[93] Pasquali R , Gambineri A. Therapy in endocrine disease: treatment of hirsutism in the polycystic ovary syndrome. Eur J Endocrinol. 2013;170(2):R75‐R90. 24272197 10.1530/EJE-13-0585

[94] Azarchi S , Bienenfeld A, Sicco KL, Marchbein S, Shapiro J, Nagler AR. Androgens in women: hormone-modulating therapies for skin disease. J Am Acad Dermatol. 2019;80(6):1509‐1521. 30312645 10.1016/j.jaad.2018.08.061

[95] Fraison E , Kostova E, Moran LJ, et al Metformin versus the combined oral contraceptive pill for hirsutism, acne, and menstrual pattern in polycystic ovary syndrome. Cochrane Database Syst Rev. 2020;8(8):CD005552. 10.1002/14651858.CD005552.pub3 PMC7437400 32794179

[96] Barrionuevo P , Nabhan M, Altayar O, et al Treatment options for hirsutism: a systematic review and network meta-analysis. J Clin Endocrinol Metab. 2018;103(4):1258‐1264. 29522176 10.1210/jc.2017-02052

